# Paradoxical Boosting of Weak and Strong Spatial Memories by Hippocampal Dopamine Uncaging

**DOI:** 10.1523/ENEURO.0469-23.2024

**Published:** 2024-05-24

**Authors:** Cintia Velazquez-Delgado, Job Perez-Becerra, Vladimir Calderon, Eduardo Hernandez-Ortiz, Federico Bermudez-Rattoni, Luis Carrillo-Reid

**Affiliations:** ^1^Instituto de Neurobiología, Universidad Nacional Autónoma de México, Juriquilla 76230, México; ^2^División de Neurociencias, Instituto de Fisiología Celular, Universidad Nacional Autónoma de México, México 04510, México

**Keywords:** animal behavior, dopamine uncaging, hippocampus, local field potentials, memory boosting, spatial memory

## Abstract

The ability to remember changes in the surroundings is fundamental for daily life. It has been proposed that novel events producing dopamine release in the hippocampal CA1 region could modulate spatial memory formation. However, the role of hippocampal dopamine increase on weak or strong spatial memories remains unclear. We show that male mice exploring two objects located in a familiar environment for 5 min created a short-term memory (weak) that cannot be retrieved 1 d later, whereas 10 min exploration created a long-term memory (strong) that can be retrieved 1 d later. Remarkably, hippocampal dopamine elevation during the encoding of weak object location memories (OLMs) allowed their retrieval 1 d later but dopamine elevation during the encoding of strong OLMs promoted the preference for a familiar object location over a novel object location after 24 h. Moreover, dopamine uncaging after the encoding of OLMs did not have effect on weak memories whereas on strong memories diminished the exploration of the novel object location. Additionally, hippocampal dopamine elevation during the retrieval of OLMs did not allow the recovery of weak memories and did not affect the retrieval of strong memory traces. Finally, dopamine elevation increased hippocampal theta oscillations, indicating that dopamine promotes the recurrent activation of specific groups of neurons. Our experiments demonstrate that hippocampal dopaminergic modulation during the encoding of OLMs depends on memory strength indicating that hyperdopaminergic levels that enhance weak experiences could compromise the normal storage of strong memories.

## Significance Statement

Increased levels of dopamine have been related to cognitive enhancement. Hippocampal dopamine elevation caused by novelty exposure has been proposed as a strategy to enhance memory based on the observation that surprising events create flashbulb memories that are remembered for long time. However, hyperdopaminergic levels could also underlie maladaptive memories, such as nondesired preservation of traumatic experiences. Our experiments show that dopamine elevation in the dorsal hippocampus during the encoding of spatial memories has paradoxical effects, while the enhancement of weak memories allows their retrieval, the dopaminergic modulation of strong memories limits the ability to modify pre-existing spatial memories by changes in the environment. We conclude that cognitive enhancement through dopamine boosting must consider diverse aspects of memory formation.

## Introduction

Spatial memories that contain information about objects in relation to the environment are essential for survival. It has been shown that the dorsal hippocampus plays an important role in the formation of spatial memories since its inactivation impairs memory retrieval (Assini et al., 2009; Iwasaki et al., 2021). Spatial memories are usually formed by recurrent experiences but in some cases could also be formed by the brief exposure to noteworthy surroundings (Duszkiewicz et al., 2019). Object location memories (OLMs) allow the measurement of changes in the environment leveraging the curiosity of mice that have been into contact with two objects to later explore for longer time a displaced object. This occurs because mice remember the previous location of the objects. OLMs could be classified in weak (short-term) or strong (long-term) depending on the time that the animals explore the objects in a familiar environment (Vogel-Ciernia and Wood, 2014; Kempadoo et al., 2016; Gálvez-Márquez et al., 2022; Bolsius et al., 2023). Accordingly, a brief exposure to two objects produces a weak memory that cannot be retrieved the next day, whereas a longer exposure to the same two objects creates a strong memory that can be retrieved 1 d later, suggesting that weak memories are prone to be lost with time and strong memories are imprinted into the brain generating robust attractor states (Rolls and Treves, 1994; Tsodyks and Sejnowski, 1995; Monasson and Rosay, 2014, 2015; Low et al., 2021).

It has been recently shown that hippocampal dopamine originating from the locus ceruleus (LC) is involved in the modulation of memories by salient events (Kempadoo et al., 2016; Takeuchi et al., 2016) and that such dopamine release in the hippocampus enhances spatial learning, suggesting that dopamine could enable the formation of associative memories (Wise, 2004; Muzzio et al., 2009). Accordingly, it has been demonstrated that LC activation during the encoding of a weak OLM allowed the retrieval of the memory 1 d later (Kempadoo et al., 2016), whereas the inactivation of LC–CA1 axons hindered the retrieval of a strong OLM (Gálvez-Márquez et al., 2022), highlighting that dopamine could modulate weak and strong OLMs.

On the other hand, it is known that dopaminergic terminals could release other neuromodulators (Tecuapetla et al., 2010; Ntamati and Luscher, 2016; Eskenazi et al., 2021) that could have significant implications in learning and memory processes. However, an exhaustive characterization of coreleased molecules by different dopaminergic nuclei to the hippocampus remains unknown (Edelmann and Lessmann, 2018) limiting the understanding of the role of dopamine elevation in hippocampal memories. Correspondingly, it has been demonstrated that LC–CA1 fibers modulate strong OLMs through the release of dopamine and noradrenaline (Gálvez-Márquez et al., 2022), whereas the same fibers modulate weak OLMs only through dopaminergic receptors (Kempadoo et al., 2016). Moreover, the dorsal hippocampus receives dopaminergic projections from the ventral tegmental area (VTA), the substantia nigra pars compacta (SNc), and the LC (Lisman and Grace, 2005; Kempadoo et al., 2016; Tsetsenis et al., 2021; Gálvez-Márquez et al., 2022). Thus, it has been proposed that the source of dopamine to the hippocampus could modulate spatial memories through distinct mechanisms. Dopamine from the VTA could be related to memory modulation by reward, whereas dopamine from the LC could be related to memory modulation by novelty (Lisman and Grace, 2005; Osorio-Gómez et al., 2023). Therefore, the effect of hippocampal CA1 dopamine elevation on spatial memories remains unclear.

Dopamine elevation in the brain has been suggested as an alternative to ameliorate cognitive deficits observed in Alzheimer's disease (Ambree et al., 2009; Shaikh et al., 2023). However, in the context of Parkinson's disease it is known that the global elevation of dopamine in the brain by the use of dopaminergic agonists produces nondesired side effects such as ʟ-DOPA induced dyskinesias, hallucinations, and compulsive behaviors (Talati et al., 2009; Voon et al., 2011; Blandini and Armentero, 2014), highlighting the need of pharmacological tools that allow the temporal and spatial control of dopamine release.

Photopharmacology represents a useful tool that limits nondesired effects observed in conventional pharmacotherapy due to poorly controlled drug release (Velema et al., 2014). Photopharmacology is based on the use of molecules that are biologically inactive due to the attachment of a photosensitive cage. Light illumination releases the attached cage allowing the interaction of the bioactive molecule with its cellular receptors. It has been highlighted that the release of molecules using caged compounds can be precisely controlled, differing from other methods such as microinjection of receptor agonists that lack spatial and temporal resolution. Moreover, the release of cage compounds occurs in the volume where the light is incident and is temporarily tied to the light parameters, whereas microinjection of receptor agonists is unavoidable linked to diffusional delays where the concentration varies from the site of the injection throughout the diffused volume (Ellis-Davies, 2007). Recently, a ruthenium-based caged compound (RuBi-Dopa) has been used to release dopamine with high spatial control allowing the study of dopamine elevation in specific brain nuclei (Araya et al., 2013; Zayat et al., 2013; Andino-Pavlovsky et al., 2017; Zamora-Ursulo et al., 2023). However, previous studies have not studied the role of hippocampal dopamine uncaging on spatial memories.

To characterize the impact of dopamine elevation in the hippocampal CA1 region, we uncaged dopamine at different stages of weak and strong OLMs. Additionally, to understand the possible neuronal population mechanisms underlying the observed behavioral effects, we measured the changes evoked by dopamine uncaging in hippocampal local field potentials (LFPs). Finally, we discussed our results following the framework of attractor models that considers spatial memories as stable network states supported by hippocampal neuronal ensembles (Rolls and Treves, 1994; Tsodyks and Sejnowski, 1995; Monasson and Rosay, 2014, 2015; Low et al., 2021).

## Materials and Methods

### Animals

Experiments were performed on C57BL/6J male mice 60–70 postnatal days before surgical procedures. Mice were housed with their littermates before and after surgical procedures. We used 138 mice for experiments and data analyses and discarded 28 animals due to failures in stereotaxic coordinates to reach the dorsal hippocampus, lack of activity during the OLM task, or object preference above 65% during the encoding stage. Mice were housed on a 12 h light/dark cycle with food and water *ad libitum*. All animal procedures were performed in accordance with the guidelines of the Institute's Bioethics Committee for the care and use of laboratory animals that comply with the standards outlined by the Guide for the Care and Use of Laboratory Animals (NIH) and the Policies on the Use of Animals and Humans in Neuroscience Research.

### Stereotaxic surgeries

Mice were anesthetized with isoflurane (1–2%) and placed in a stereotaxic system (Stoelting). All procedures were performed in sterile conditions. Respiratory rate and tail pinch reflex were monitored along the surgery. For all mice a custom designed stainless steel head plate was attached to the skull using dental cement. Experiments were performed ∼1 week after head plate implantation. After surgeries, mice were handed and exposed to head fixation conditions for several days before the task protocol to avoid possible effects in the OLM task caused by stress. On all mice used for the OLM task, a 0.5 mm craniotomy was performed bilaterally on top of the dorsal hippocampus (AP: 2.5 mm; ML: ±1.5 mm; DV: −1.4 mm) to stereotaxically insert a cannula (22 gauge; 5 mm long) that was used to locally inject RuBi-Dopa (Abcam) into the CA1 region and subsequently introduce a fiber-optic cannula for light uncaging (400 µm diameter, 0.39 NA, Thorlabs). On a different group of mice, a microdialysis cannula to measure hippocampal DA and NA levels and a cannula for the fiber-optic and RuBi-Dopa injection were implanted, with an angle of 25° between them, such as that both tips of the cannulae converged. The cannulae used have a removable dummy protective cap to avoid clogging. During surgeries, eyes were moisturized with eye ointment. For 5 d after surgery, mice received subcutaneously 0.5 ml of saline/glucose (4%) solution to prevent dehydration.

### Object location memory task

The OLM paradigm is ideal to study dopaminergic modulation because it is based on the natural exploratory behavior of animals without relying on rewards or punishments (Vogel-Ciernia and Wood, 2014) that could alter dopamine levels. The OLM was evaluated in an open-field acrylic cylinder (30 cm diameter and 45 cm height). A black and white striped contextual cue (5 × 30 cm, with 1 cm stripes) was glued to the top side of the wall (observed from above). Two identical objects were used. The displaced object in the retrieval stage was selected randomly. For visual purposes, all figures show the displaced object on the right side. Animals were habituated to the open-field cylinder for 3 consecutive days, allowing its spontaneous exploration without objects for 10 min (habituation). One day later, mice were introduced into the same open-field cylinder containing two identical objects aligned in a horizontal line (observed from above). For the weak OLM task, mice were allowed to explore both objects for 5 min, whereas for the strong OLM task, in a different group of animals, mice were allowed to explore the same two objects for 10 min during the encoding stage. One day later, mice were introduced into the open-field cylinder containing the same two objects but with one object displaced vertically (novel location) toward the bottom side of the wall (observed from above). For both, weak and strong OLMs mice were allowed to explore the two objects for 5 min during the retrieval stage.

For some behavioral experiments, the D1-like receptor antagonist SCH 23390 (1.125 µg/1 µl; Sigma-Aldrich) was locally injected in the hippocampal CA1 region according to previous reports (Kempadoo et al., 2016). In such experiments, mice were injected bilaterally (0.5 µl per side) before dopamine uncaging.

### Automatic tracking of mice movement and object exploration

Mice were recorded with a commercial USB camera (640 × 360 pixels, 30 Hz acquisition rate). Images correspond to a top view of the open-field cylinder where two identical objects were fixed. After recordings were finished, videos were cropped to the diameter of the cylindrical area. The nose, headplate, back, tail, the corners of both objects, and the cylinder top, bottom, right, and left edges (observed from the top) were automatically detected using DeepLabCut (DLC) version 2.3.5 (Mathis et al., 2018). The network architecture MobileNet version 2 1.0 (Mathis et al., 2021) was trained in a Windows 11 PC (Dell OptiPlex 7090 with Intel Core i7 CPU 2.90 GHz and 40 GB RAM) using the default DLC settings. A total of 740 frames were marked achieving training and test errors of 2.54 and 4.26 pixels, respectively, after 500,000 iterations. Custom scripts were written in MATLAB (MathWorks) to analyze the coordinates detected by DLC of the objects and the mouse body. The distance of the nose to the objects was measured to determine exploration time. An object exploration was considered when the nose distance to any object was between 0 and 2 cm, without any other body part overlapping the object. In this way the recognition index for each object was computed using the total number of frames that mice explored one object divided by the total number of frames that mice explored both objects.

### Dopamine uncaging

RuBi-Dopa (1.5 µl; 300 µM; 0.3 µl/min; Abcam) was injected locally into the left and right hippocampi through implanted cannulae. For RuBi-Dopa injections, mice were placed on a custom designed treadmill system. An injector needle connected to an infusion pump (Fusion 200, Chemix) was inserted through the cannulae. After RuBi-Dopa injection, the needle was removed, and diffusion was allowed for 10 min. Fiber-optic cannulae attached to a compatible fiber optic and connected to a blue LED (470 nm) were inserted through the injection cannulae, and RuBi-Dopa was irradiated with light for 10 min using a LED controller (CD2100, Thorlabs, 20 Hz, 470 nm, 20% duty cycle, 4 mW). Such blue light irradiation with a fiber optic of 400 µm diameter produces minimal thermal effects that does not affect neuronal activity (Stujenske et al., 2015). The fiber-optic cannulae were removed after dopamine uncaging, and the animals were placed on the open-field arena after 10 min of dopamine uncaging.

### Microdialysis and electrophoresis

A microdialysis probe (1 mm CMA-7, 6 kDa, CMA) connected to an infusion pump was inserted through the cannula placed in the right hippocampus of anesthetized mice (isoflurane; 2%) on a stereotaxic apparatus. Artificial cerebrospinal fluid (ACSF) was perfused at 0.25 µl/min. After the probe insertion, we waited for 1 h to avoid artifacts evoked by mechanical manipulation. RuBi-Dopa was injected for 5 min, and 10 min was allowed for diffusion. Three samples were collected every 20 min to calculate the baseline levels of dopamine (DA) and noradrenaline (NA). Then RuBi-Dopa was irradiated for 10 min (20 Hz, 470 nm, 20% duty cycle, 4 mW). After dopamine uncaging, five samples were collected every 20 min. After collection, samples were frozen (−80°C) and then analyzed by electrophoresis. Capillary electrophoresis was used to quantify neurotransmitter concentration. Microdialysis samples were derivatized by adding 6 μl of 3-(2-furoyl)-quinoline-2-carboxaldehyde (FQ, 16.67 mM, Molecular Probes; Invitrogen). This reaction was catalyzed by 2 μl KCN (24.5 mM) in borate buffer (10 mM), pH 9.2 in the presence of 1 μl of an internal standard (0.075 mM, *O*-methyl-ʟ-threonine; Fluka). The mixture was incubated in the dark for 15 min at 65°C. Subsequently, the neurotransmitters were detected with laser-induced fluorescence within a capillary electrophoresis system (P/ACE MDQ, Beckman Coulter). Compound separation was based on a micelle electrokinetic chromatography method. Samples were hydrodynamically injected into the capillary system at 0.5 psi for 5 s. Separation occurred in the presence of a buffer (borates 35 mM, sodium dodecyl sulfate 25 mM, and 13% methanol HPLC grade, pH 9.6) at 20 kV. Neurotransmitters migrate until they are separated and detected by fluorescence using a laser-induced fluorescence detection device (488 nm). Signals were depicted as electropherograms that later were analyzed using 32Karat TM8.0 software (Beckman Coulter). Neurotransmitters were identified and quantified by comparison with the standard electropherogram pattern (DA and NA standards).

### Electrophysiology

To perform LFP recordings of hippocampal activity a 3 mm diameter craniotomy over the right hippocampus was performed in anesthetized mice (isoflurane 1–2%) placed in a stereotaxic system 1 week after headplate implantation. After craniotomy, mice were head-fixed on a custom-designed treadmill and allowed to wake up. An injector attached to a fiber-optic cannula connected to a fiber optic and a blue LED (470 nm) was inserted at 20° from the vertical line on the dorsal hippocampus. A silicon probe (NeuroNexus, A4 × 4-tet-5 mm) was inserted vertically (AP: 2.5 mm; ML: ±1.5 mm; DV: −1.4 mm) until it converged with the fiber-optic cannula on CA1. LFPs were acquired with OmniPlex Neural recording data acquisition system (Plexon) and low-pass filtered (<300 Hz).

The power spectrum was parameterized by fitting periodic and aperiodic components as it has been shown (Donoghue et al., 2020). The mean power for theta oscillations (4–8 Hz) was calculated from the subtracted periodic component of the power spectrum. Data analyses were restricted to stationary states to avoid possible effects on the theta band caused by running.

### Analyses and statistical methods

We did not use statistical power analysis to determine the number of animals used in each experiment. We determined the sample size following previous publications using a similar OLM task. All values in the text indicate mean ± SD. Male mice littermates were randomly assigned to experimental groups before surgeries. Experimental data were collected not blinded to experimental groups. MATLAB R2021b (MathWorks) was used for data analysis. Statistical tests were done in GraphPad Prism. Statistical details of each experimental group can be found in figure legends. Two-tail tests were performed in all behavioral and electrophysiological experiments. Data are presented as whisker boxplots displaying median, interquartile, and range values. Since the recognition index of the object in the novel location is dependent on the recognition index of the object in the familiar location, we tested the recognition index against 50% chance of exploration. Recognition indexes for both novel locations and familiar locations are presented in the figures.

## Results

### Characterization of weak and strong OLMs

**Figure 1.**
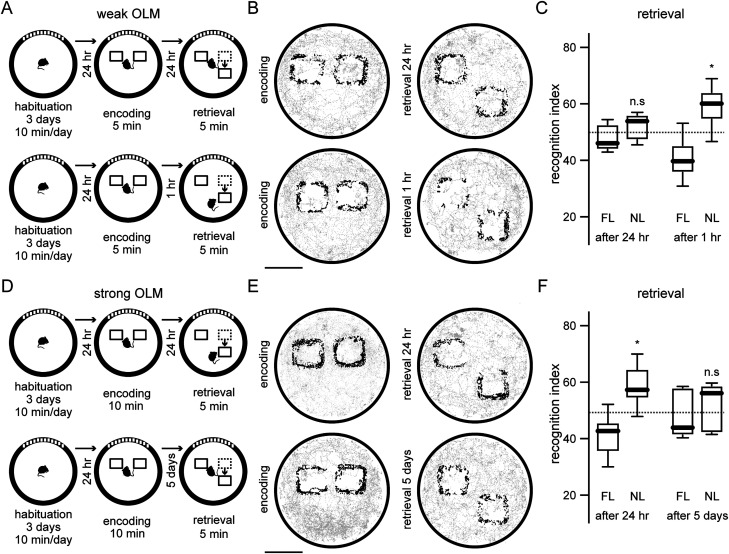
Weak and strong OLM tasks. ***A***, Schematic representation of the implementation of a weak OLM task in two different experimental groups. Group 1: retrieval stage at 24 h (top). Group 2: retrieval stage after 1 h (bottom). ***B***, Spatial maps of representative movement trajectories automatically identified during encoding (left) and retrieval (right) for the same groups from ***A***. Black dots represent object exploration. Gray dots depict nonexploration events. Scale bar, 10 cm. ***C***, Mice explored at chance levels (50%) the familiar location (FL) and the novel location (NL) during the retrieval stage 24 h later (*p* = 0.2188) indicating that the spatial memory was not formed, whereas mice explored more the displaced object 1 h after the encoding stage (*p = 0.0312), indicating the formation of a short-term memory. ***D***, Schematic representation illustrating the timeline of a strong OLM task in two different experimental groups where the retrieval stage was performed 24 h (top) or 5 d after (bottom) the encoding stage. ***E***, Movement trajectory of two representative mice for a strong OLM in the two experimental groups from ***D***. Scale bar, 10 cm. ***F***, Mice explored more than expected by chance (50%) the displaced object after 24 h (*p = 0.0312) indicating that the spatial memory was formed, whereas mice explore at chance levels the displaced object 5 d later (*p* = 0.3750) indicating that the long-term memory was lost. For all experimental groups, one-sample Wilcoxon signed rank test was used (*n* = 7 mice for each group).

To characterize weak or strong OLMs, we considered that the exploration time of two identical objects in a familiar environment during the encoding stage of memory could have distinct effects on the retrieval stage 1 d later as it has been previously suggested (Vogel-Ciernia and Wood, 2014). It is known that when mice remember the location of the objects, the displacement of one object promotes the preferential exploration of the novel location (Murai et al., 2007). Accordingly, for the weak OLM protocol, mice were allowed to examine the objects for 5 min during the encoding stage and 24 h later one of the objects was moved (Fig. 1*A*, top). We automatically tracked the trajectory of each mouse (Materials and Methods) during the encoding and retrieval stages (Fig. 1*B*, top) and observed that the recognition index for the object in the novel location during the retrieval stage was similar against 50% chance of exploration (Fig. 1*C*; recognition index novel location after 24 h: 52.47 ± 4.515), indicating a lack of memory. To corroborate that the weak OLM protocol induced short-term memory (Murai et al., 2007), in a different experimental group, mice were allowed to explore the objects for 5 min during the encoding stage and 1 h later one of the objects was displaced (Fig. 1*A*, bottom). Automatically tracking the trajectory of each mouse (Fig. 1*B*, bottom), we observed that during the retrieval stage, mice explored the displaced object more than expected by chance (Fig. 1*C*; recognition index novel location after 1 h: 59.00 ± 7.161), confirming that the weak OLM protocol creates a short-term memory. On the other hand, for the strong OLM protocol mice were allowed to explore the objects for 10 min and 24 h later one of the objects was moved (Fig. 1*D*,*E*, top). Under these conditions, mice explored more the displaced object during the retrieval stage than expected by chance (Fig. 1*F*; recognition index novel location after 24 h: 58.52 ± 7.163), showing long-term memory formation. To investigate if the long-term memory can be retrieved several days later, a different group of mice was trained with the strong OLM protocol but performed the retrieval stage 5 d later instead of 1 d later (Fig. 1*D*,*E*, bottom). We observed that mice interacted equally to chance levels with the displaced object during the retrieval stage (Fig. 1*F*; recognition index novel location after 5 d: 51.82 ± 8.31) indicating that the long-term memory is lost after 5 d. These experiments confirm that a brief exposure to two objects in a familiar environment creates a weak OLM that cannot be retrieved 1 d later, whereas a longer exposure to the same two objects generates a strong OLM that can be retrieved 1 d later.

### Hippocampal dopamine elevation during the encoding of weak and strong spatial memories

**Figure 2.**
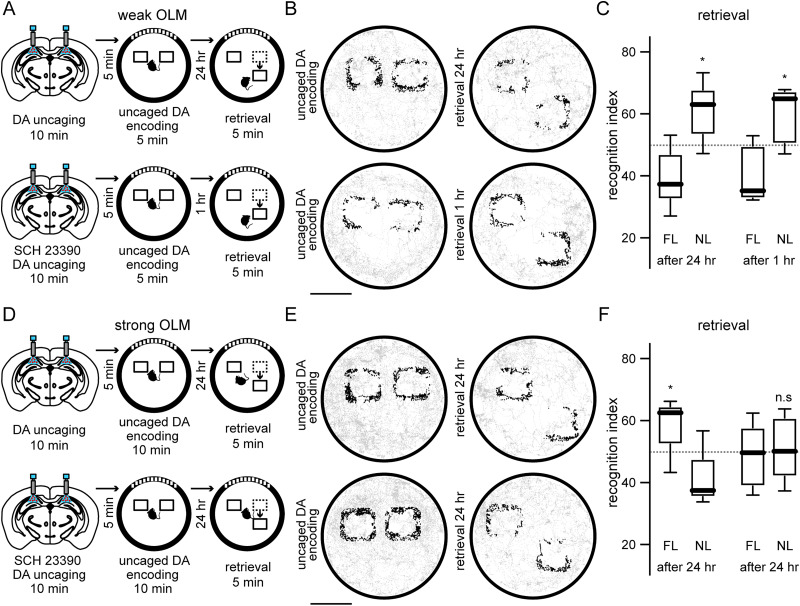
Hippocampal dopamine elevation during encoding of OLMs. ***A***, Experimental timeline of bilateral dopamine uncaging before the encoding of a weak OLM in two different experimental groups. Group 1: retrieval stage after 24 h (top). Group 2: retrieval stage after 1 h in the presence of the D1 like receptor antagonist SCH 23390 (bottom). ***B***, Spatial maps of the trajectory of two representative mice for a weak OLM in the experimental conditions shown in ***A***. Scale bar, 10 cm. ***C***, Mice explored more the displaced object (*p = 0.0312) after 24 h indicating that dopamine elevation enhanced weak memory retrieval. On the other hand, mice explored more the displaced object (*p = 0.0469) after 1 h in the presence of SCH 23390 indicating that short-term memory after 1 h is independent of dopamine action. ***D***, Schematic representation of hippocampal dopamine uncaging before the encoding of a strong OLM in two different experimental groups. Group 1: retrieval stage after 24 h (top). Group 2: retrieval stage after 24 h in the presence of the D1-like receptor antagonist SCH 23390 (bottom). ***E***, Spatial maps of the trajectory of two representative mice for a strong OLM in the experiments shown in ***D***. Scale bar, 10 cm. ***F***, Mice preferred the familiar location (*p = 0.0469) in the retrieval stage 24 h after dopamine elevation suggesting that the mechanism for increased exploration of a displaced object was compromised. On the other hand, mice explored equally the novel location compared with chance levels (*p* = 0.9375) 24 h after encoding in the presence of SCH 23390, indicating that long-term memory depends on dopaminergic action. For all experimental groups one-sample Wilcoxon signed rank test was used (*n* = 7 mice for each group).

It has been proposed that learning involves multiple stages such as encoding, consolidation, and retrieval (Squire and Zola, 1996; Guskjolen and Cembrowski, 2023). Because dopamine could modulate such stages differentially, we studied the effect of local hippocampal dopamine elevation on weak and strong memories at different stages. Using in vivo microdialysis with electrophoretic detection (Materials and Methods), we quantified the extracellular levels of dopamine (DA) and noradrenaline (NA) and observed that our uncaging protocol using blue light (470 nm, 20 Hz, 4 mW, 20% duty cycle) produced dopamine increase in the hippocampus, as expected, keeping noradrenaline levels unaffected, and that such elevation lasted for at least 40 min [DA basal (a.u): 6.656 ± 2.206; DA 40 min after uncaging: 16.87 ± 3.02; *p = 0.0312; *n* = 5 mice; Wilcoxon matched-pairs signed rank test; NA basal (a.u): 4,752 ± 2,370; NA 40 min after uncaging: 4,586 ± 2,903; *p* = 0.3125; *n* = 5 mice; Wilcoxon matched-pairs signed rank test]. As a control experiment, in a different group of mice, we used the same light delivery protocol but using red light (617 nm) that is unable to uncage ruthenium caged compounds. We observed that the extracellular levels of dopamine and noradrenaline remained unaffected using red light (617 nm) in the hippocampus, as expected [DA basal (a.u): 2.672 ± 1.552; DA 40 min after red illumination: 3.248 ± 1.165; *p* = 0.3125; *n* = 5 mice; Wilcoxon matched-pairs signed rank test; NA basal (a.u): 2,348 ± 2,464; NA 40 min after red illumination: 2,369 ± 1,569; *p* = 0.5; *n* = 5 mice; Wilcoxon matched-pairs signed rank test], indicating that during the encoding stage hippocampal dopamine levels were elevated by successful blue light uncaging. To characterize the outcome of hippocampal dopamine elevation during the encoding stage of a weak OLM, we uncaged dopamine bilaterally for 10 min into the dorsal hippocampus of head fixed mice (Materials and Methods) using fiber-optic cannulae attached to a blue LED (470 nm). Five minutes after the uncaging protocol, mice were placed in a familiar environment and allowed to explore the two identical objects for 5 min. Then, 24 h later mice were exposed to the same environment for 5 min but with one object displaced (Fig. 2*A*,*B*, top). Under these conditions, mice spent more time in the displaced object during the retrieval stage (Fig. 2*C*; recognition index novel location after 24 h: 60.28 ± 9.152), demonstrating that hippocampal dopamine elevation enhanced weak memories. Such weak memory enhancement was not a product of increased object exploration caused by dopamine elevation since the interaction time with both objects during the encoding stage was similar to control conditions without dopamine uncaging [*p* = 0.9015; *n* = 7 mice; Mann–Whitney test; interaction time during encoding (seconds): no uncaging: 23.93 ± 12.96; uncaged DA: 24.35 ± 10.99 s]. To further investigate if the creation of short-term memory evoked by the weak OLM protocol is dependent on dopamine modulation, on a different group of mice, we uncaged dopamine during the encoding stage of a weak memory in the presence of a D1-like receptor antagonist (Fig. 2*A*,*B*, bottom). We observed that 1 h after encoding mice spent more time in the displaced object during the retrieval stage (Fig. 2*C*; recognition index novel location after 1 h: 59.58 ± 8.78), demonstrating that short-term memory is independent of dopamine-related mechanisms. Moreover, to disregard that the enhancement of the weak memory was an artifact of light irradiation, in a different group of mice, we performed the weak OLM protocol but substituting RuBi-Dopa by saline injection. Under such conditions, the weak OLM protocol was not able to enhance spatial memory after 24 h (*p* = 0.9375; *n* = 7 mice; recognition index novel location after 24 h: 48.93 ± 14.02; one-sample Wilcoxon signed rank test) confirming that our photostimulation protocol did not affect neuronal activity (see Materials and Methods). On a different group of mice, we studied the effect of dopamine elevation during the encoding stage of a strong OLM using the same uncaging protocol. Then, 24 h later mice were exposed to the same environment for 5 min but with one object displaced (Fig. 2*D*,*E*, top). We observed that mice interacted more time than expected by chance with the nondisplaced object during the retrieval stage (Fig. 2*F*; recognition index familiar location: 58.93 ± 8.184), revealing that hippocampal dopamine elevation promoted the exploration of the familiar location over the novel location in strong OLMs. Such familiar location fixation was not a product of changes in object exploration caused by dopamine elevation since the interaction time with both objects during the encoding stage was similar to control conditions without dopamine uncaging [*p* = 0.62; *n* = 7 mice; Mann–Whitney test; interaction time during encoding (seconds): no uncaging: 65.59 ± 10.05; uncaged DA: 65.59 ± 13.7]. To investigate if the familiar location fixation was a direct effect of dopaminergic action, in a different group of mice we uncaged dopamine during the encoding of a strong OLM in the presence of a D1 like receptor antagonist (Fig. 2*D*,*E*, bottom). We observed that mice explored similarly to chance levels the displaced object during the retrieval stage 1 d later (Fig. 2*F*; recognition index novel location: 50.56 ± 10.26), indicating that long-term memory engages dopamine-dependent processes. To further investigate if the familiar location fixation evoked by dopamine elevation during the encoding stage of a strong OLM was related to abnormally boosted memory, we performed experiments on a different group of mice, where we waited 5 d for the retrieval stage instead of 1 d. Under these conditions, we observed that mice spent more time than expected by chance in the displaced object during the retrieval stage, demonstrating that hippocampal dopamine elevation over strengthened strong memories (Rossato et al., 2009), making them available for longer time than usual (*p = 0.0469; *n* = 7 mice; recognition index novel location after 5 d: 56.66 ± 6.38; one-sample Wilcoxon signed rank test). These experiments show that dopamine elevation throughout the encoding stage of OLMs boosted memory formation. In the case of weak memories, such enhancement allowed the preservation of a memory that is normally lost after few hours, whereas in the case of strong memories their increased enhancement promoted the fixation of the familiar location 1 d later, resembling the observation of spatial memory impairment by saturation of long-term potentiation (Moser et al., 1998) but allowed the successful retrieval of the strong OLM 5 d later, indicating the boosted imprinting of strong memories.

### Impairment of strong memories by hippocampal dopamine uncaging after encoding

**Figure 3.**
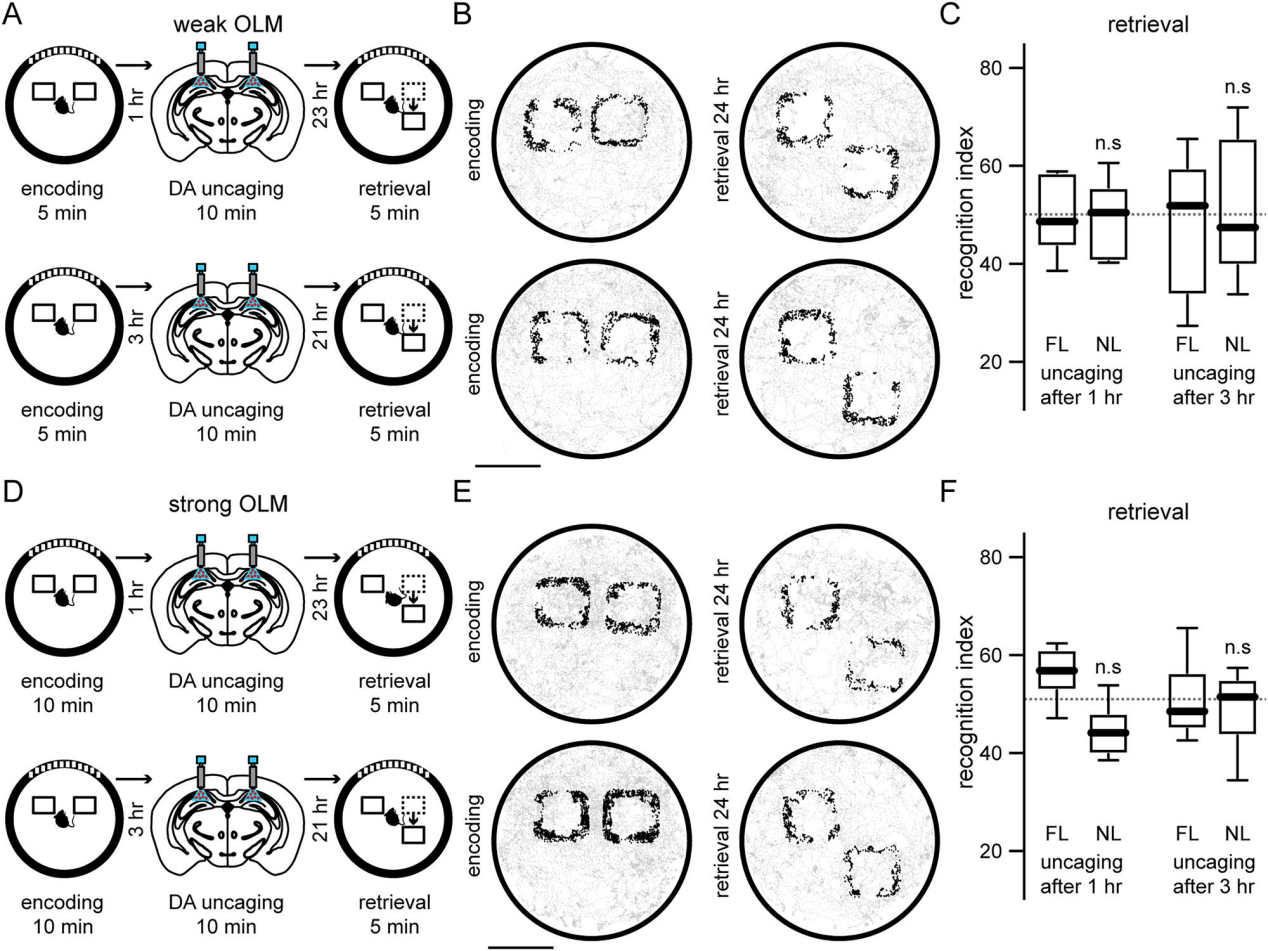
Hippocampal dopamine uncaging after encoding of OLMs. ***A***, Schematic representation of hippocampal bilateral irradiation of RuBi-Dopa 1 h (top) or 3 h (bottom) after the encoding of a weak OLM in two different experimental groups. ***B***, Spatial maps of the trajectory of two representative mice for a weak OLM in the same experimental conditions as in ***A***. Scale bar, 10 cm. ***C***, Mice explored equally to chance levels the displaced object during the retrieval stages for both experimental conditions (DA after 1 h of encoding: *p* = 0.9999; DA after 3 h of encoding: *p* = 0.9375), indicating that dopamine elevation after encoding was not able to enhance weak memories. ***D***, Schematic representation of hippocampal dopamine uncaging 1 h (top) or 3 h (bottom) after the encoding of a strong OLM in two different groups of mice. ***E***, Spatial maps of the trajectory of two representative mice for a strong OLM in the experimental conditions shown in ***D***. Scale bar, 10 cm. ***F***, Mice explored equally to chance levels the displaced objects during the retrieval stage for both experimental conditions (DA after 1 h of encoding: *p* = 0.0781; DA after 3 h of encoding: *p* = 0.8125), indicating that dopamine elevation after encoding of a strong OLM interfered with the mechanisms underlying the increased exploration of novel locations. For all experimental groups, one-sample Wilcoxon signed rank test was used (*n* = 7 mice for each group).

Our previous experiments suggest that dopamine elevation after the encoding stage could have different effects on weak or strong memories, since the formation of short-term memories was independent of D1-like receptor activation. To investigate if there is a time window after encoding where dopamine elevation could enhance weak OLMs, we perform the weak OLM protocol in a different group of mice where we uncaged dopamine 1 h after the encoding of an OLM (Fig. 3*A*,*B*, top). We observed that at such time window dopamine elevation was not able to enhance weak OLMs (Fig. 3*C*; recognition index novel location for DA uncaging after 1 h: 49.78 ± 7.486). It has been shown that short-term OLMs where mice explored two identical objects for 5 min during the encoding stage cannot be retrieved successfully 3 h later (Murai et al., 2007), so we investigated if hippocampal dopamine elevation after encoding could enhance weak memories that are no longer available. We performed the same weak OLM training as before, in a different group of mice, but uncaging dopamine 3 h after the encoding stage. Then, 21 h later mice were exposed to the same environment for 5 min but with one object displaced (Fig. 3*A*,*B*, bottom). We observed that mice explored equally to chance levels the displaced object during retrieval stage (Fig. 3*C*; recognition index novel location for DA uncaging after 3 h: 51.33 ± 14.08), showing that hippocampal dopamine elevation 3 h after encoding was not able to rescue nonretrievable OLMs. On two different groups of mice, we studied the effect of dopamine elevation during the consolidation stage (1 or 3 h after encoding) of a strong OLM (Fig. 3*D*,*E*). We observed that mice explored similarly to chance levels the displaced objects during their corresponding retrieval stages (Fig. 3*F*; recognition index novel location DA uncaging after 1 h: 44.20 ± 5.557; recognition index novel location DA uncaging after 3 h: 48.25 ± 8.02), demonstrating that hippocampal dopamine elevation during the consolidation stage impaired the OLM task. These experiments suggest that dopamine elevation in the dorsal hippocampus after the encoding stage mainly modulate newly formed synaptic connections (Asok et al., 2019). We conclude that dopamine elevation after the encoding stage cannot enhance short-term spatial memories that are independent of dopaminergic mechanisms, whereas dopamine elevation after the encoding stage impaired long-term spatial memories.

### Lack of modulation of weak and strong OLMs by hippocampal dopamine elevation during retrieval

**Figure 4.**
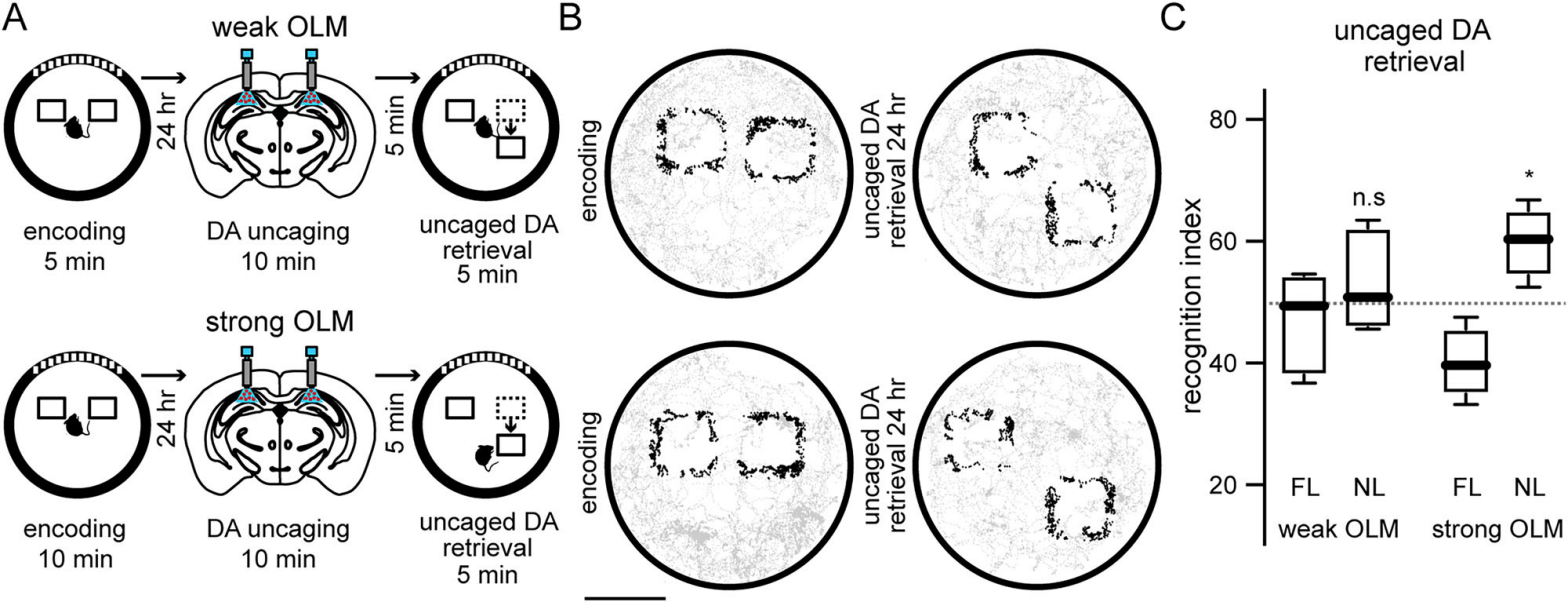
Hippocampal dopamine elevation during retrieval of OLMs. ***A***, Experimental timeline of hippocampal bilateral irradiation of RuBi-Dopa before the retrieval of a weak (top) or a strong (bottom) OLM in two different experimental groups. ***B***, Spatial maps of the trajectory of two representative mice for the same experimental conditions as ***A***. Scale bar, 10 cm. ***C***, Mice explored equally to chance levels the displaced object in the weak OLM protocol (*p* = 0.6875), indicating that dopamine elevation during retrieval was not able to rescue lost memories. On the other hand, during the retrieval stage of strong OLMs mice explored more the novel location (*p = 0.0156), indicating a lack of effect of dopamine elevation during retrieval. For all experimental groups, one-sample Wilcoxon signed rank test was used (*n* = 7 mice for each group).

It has been shown that inhibition of LC–CA1 terminals during the retrieval stage of a strong OLM impedes the increased exploration of the displaced object (Gálvez-Márquez et al., 2022), suggesting that dopamine is necessary for the successful expression of OLMs. However, the outcome of hippocampal dopamine elevation during the retrieval stage of weak or strong OLMs remains unknown. Therefore, in two different groups of mice, we uncaged dopamine in the dorsal hippocampus 5 min before the retrieval stage of a weak OLM or a strong OLM, respectively (Fig. 4*A*,*B*). Under these conditions, mice explored equally to chance levels the displaced object during the retrieval stage of a weak OLM (Fig. 4*C*; recognition index novel location weak OLM: 52.7 ± 7.421), whereas during the retrieval stage of a strong OLM, mice still had a preference to explore more than expected by chance the displaced object (Fig. 4*C*; recognition index novel location strong OLM: 60.09 ± 5.181), indicating that dopamine elevation during retrieval does not affect weak or strong memories. Our experiments demonstrate that hippocampal dopamine elevation spares pre-existing spatial memories.

### Enhancement of hippocampal theta oscillations by dopamine uncaging

**Figure 5.**
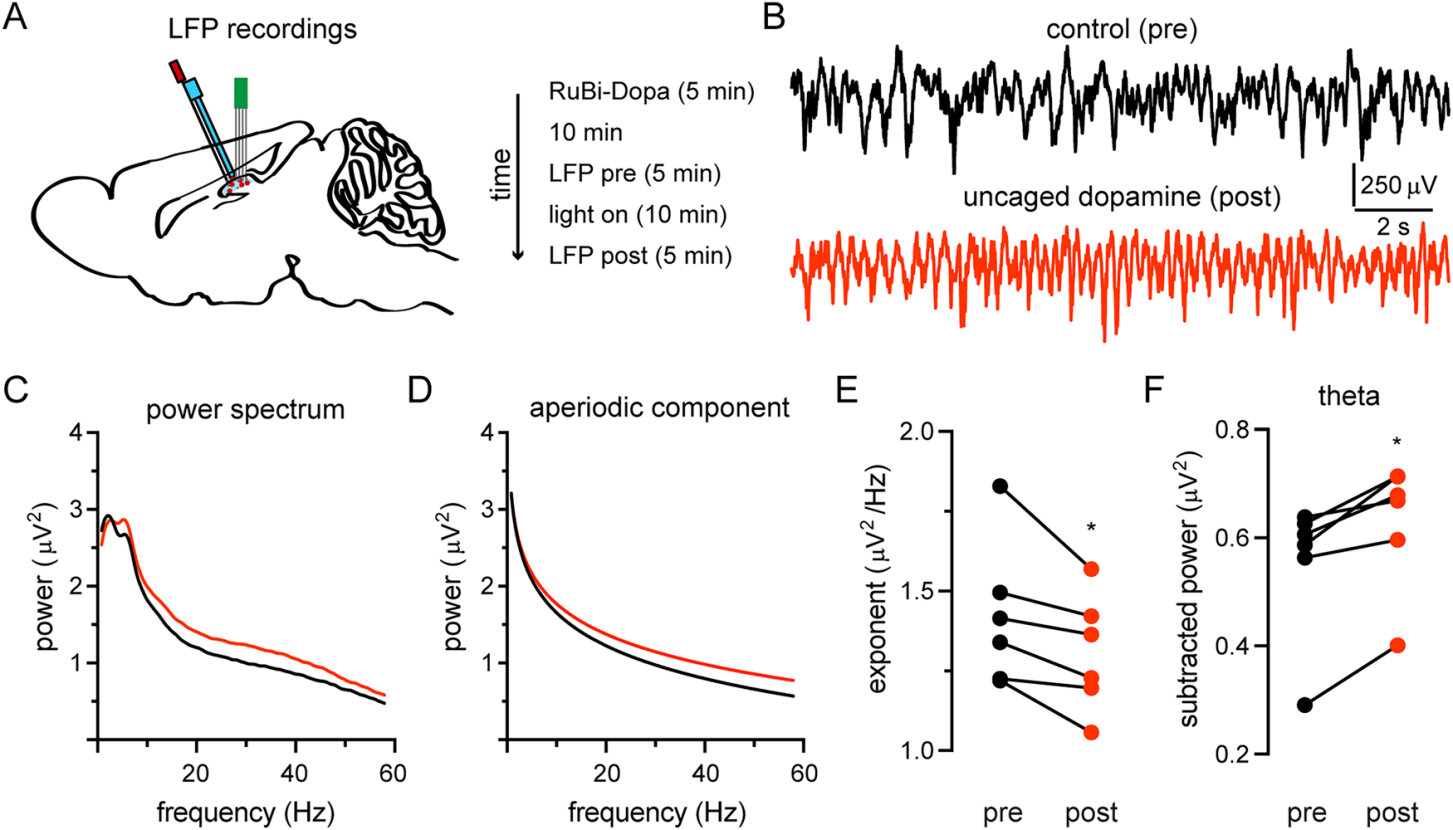
Effect of hippocampal dopamine uncaging on LFPs. ***A***, Schematic representation of hippocampal dopamine uncaging and LFP recordings. ***B***, Hippocampal LFPs recorded before (black) and 10 min after local dopamine uncaging (red). ***C***, Power spectrum of hippocampal LFPs before (black) and after (red) dopamine uncaging. Note the wideband increase of the power spectrum reflected as a positive shift caused by hippocampal dopamine elevation. ***D***, Parameterization of the power spectrum into aperiodic and periodic components before (black) and after (red) dopamine uncaging. Note a positive shift in the aperiodic component caused by uncaged dopamine that is more prominent after 10 Hz. ***E***, The exponent of the aperiodic component was significantly decreased (*p = 0.0312). ***F***, The subtraction of the aperiodic component from the power spectrum shows that theta oscillations (4–8 Hz) were enhanced after dopamine uncaging suggesting the entrainment of neuronal ensembles (*p = 0.0312). Wilcoxon matched-pairs signed rank test (*n* = 6 mice).

It has been proposed that the activation of hippocampal neuronal ensembles underlies theta oscillations and information storage (Buzsaki, 2002; Manns et al., 2007). Moreover, it has been demonstrated that the disruption of theta oscillations impairs spatial learning and that the restoration of theta oscillations rescues spatial learning (McNaughton et al., 2006). However, the characterization of changes in hippocampal theta oscillations mediated by dopamine elevation remains unknown. So far, our experiments suggests that hippocampal dopamine elevation could boost spatial memories by changing the interaction of internal activity patterns with information coming from the environment, indicating that theta oscillations could be modulated by dopamine. Thus, we characterized the changes on population electrical activity recording LFPs in awake mice (Fig. 5*A*) during stationary states (see Materials and Methods) before and after hippocampal dopamine uncaging (Fig. 5*B*). We observed a wideband enhancement of CA1 oscillations evoked by dopamine elevation that was reflected as a positive shift in the amplitude of the power spectrum (Fig. 5*C*). To further investigate if specific frequency bands were modulated by dopamine elevation, we separated the power spectrum into periodic and aperiodic components (Donoghue et al., 2020). We observed a significant increase in the aperiodic component after dopamine uncaging (Fig. 5*D*) that was reflected as a decreased exponent (Fig. 5*E*; exponent before DA uncaging: 1.421 ± 0.2267; after dopamine uncaging: 1.306 ± 0.1821). On the other hand, after subtracting the power spectrum from the aperiodic component, we observed a significant enhancement of the theta band (4–8 Hz; Fig. 5*F*; subtracted theta power before DA uncaging: 0.5519 ± 0.1307; after dopamine uncaging: 0.6286 ± 0.1195). These results indicate that hippocampal dopamine elevation increased synaptic activity and potentiated the entrainment of hippocampal neuronal ensembles to theta oscillations.

Both actions could produce two different effects on hippocampal function depending on memory strength. On the one hand, during the encoding stage of weak memories, the increased activity of hippocampal neuronal ensembles related to the spatial memory results in long-term storage of otherwise lost ensembles. On the other hand, during the encoding stage of strong memories, the boosted entrainment of robust neuronal ensembles could evoke changes in synaptic connectivity that result in the decreased exploration of the displaced object.

## Discussion

Our experiments demonstrate that dopamine elevation in the dorsal hippocampus during the encoding of weak or strong OLMs boosts memory formation producing a dichotomous outcome. While the enhancement of weak OLMs allowed the retrieval of otherwise lost spatial memories, the abnormal boosting of strong memories promoted the preference for familiar object locations. Furthermore, hippocampal LFP recordings showed that dopamine uncaging increased noise levels and hippocampal theta oscillations, supporting the view that the entrainment of neuronal ensembles produces memory boosting.

### Dopaminergic modulation of weak spatial memories

It has been shown that short-term memories and long-term memories have different molecular mechanisms (Argyrousi et al., 2020). Accordingly, we observed that hippocampal application of a D1-like receptor antagonist during the encoding stage did not affect the successful retrieval of weak OLMs 1 h later (Fig. 2). However, hippocampal dopamine elevation during the encoding stage of weak OLMs allowed their successful retrieval 1 d later (Fig. 2), confirming the previously reported enhancement of weak spatial memories by hippocampal dopamine elevation (Kempadoo et al., 2016). The enhancement of weak OLMs by hippocampal dopamine elevation during the encoding stage is consistent with previous findings indicating that D1-like receptor activation inhibits the depotentiation of CA1 synapses raising the possibility to retain memories by delaying their erasure (Otmakhova and Lisman, 1998). While our results show that dopamine elevation modulates weak memories during encoding, we observed that dopamine elevation after the encoding stage or at the retrieval stage was not able to enhance weak memories (Figs. 3, 4) supporting the view that dopamine must be present at the time of memory formation to affect early long-term potentiation at CA1 synaptic connections (Otmakhova and Lisman, 1996). Our results indicate that after short-term spatial memories are lost, they cannot be recovered by dopaminergic modulation.

### Dopaminergic modulation of strong spatial memories

It has been proposed that dopamine elevation promotes the enhancement of memory formation (Lisman and Grace, 2005; Lisman et al., 2011; Duszkiewicz et al., 2019). Surprisingly, the effect of hippocampal dopamine elevation on strong OLMs has not been documented. Our experiments showed that the blockade of D1-like receptors during the encoding stage of strong OLMs impaired their retrieval 1 d later (Fig. 2) indicating that dopaminergic mechanisms are necessary for long-term memories.

Interestingly, dopamine elevation during the encoding of strong OLMs increased the preference for the familiar object location over the novel object location 1 d later (Fig. 2). Such preference should not be interpreted as a memory impairment since our experiments show the correct retrieval of strong OLMs formed under hyperdopaminergic conditions 5 d later, demonstrating the formation of an abnormally stable memory. Accordingly, it has been suggested that hyperdopaminergic levels could isolate CA1 from sensory feedback coming from the entorhinal cortex (Otmakhova and Lisman, 1999) while at the same time could reinforce the activation of neuronal activity patterns coming from CA3 (Otmakhova and Lisman, 1998). Thus, during the retrieval of an abnormally enhanced strong memory, neuronal ensemble activity patterns could be bias to information coming from CA3 (Kesner and Rolls, 2015), suggesting that the boosted reactivation of stored ensembles underlies the preference to visit the familiar object location by dampening the salience of the novel object location. Consistently, it has been shown that the constant replaying of a learned trajectory in stationary conditions predicts the further navigation of such trajectory (Pastalkova et al., 2008). Our experiments suggest that the repeated reactivation of neuronal ensembles representing the familiar object location using two-photon optogenetics (Carrillo-Reid et al., 2019; Robinson et al., 2020) could lead to the preference for the familiar object location over the novel object location. It is important to highlight that the preference for the familiar object location 1 d after the encoding of strong OLMs during hyperdopaminergic conditions agrees with the creation of an abnormally enhanced spatial memory and the possible saturation of long-term potentiation (Moser et al., 1998).

Moreover, we observed that dopamine elevation during the consolidation stage of strong OLMs impaired their retrieval 1 d later (Fig. 3), suggesting the nonspecific entrainment of neuronal ensembles that interfere with the original memory (Colgin et al., 2008). Finally, our experiments show that the retrieval of fully formed spatial memories was not affected by hippocampal dopamine elevation (Fig. 4).

### Entrainment of neuronal ensembles by hippocampal dopamine uncaging

It has been suggested that memory engrams are composed by different neuronal ensembles representing specific memory features (Ghandour et al., 2019; Carrillo-Reid, 2022; Marks et al., 2022). In this context, neuronal ensembles are groups of neurons with synchronous activity that could be related to different parts of spatial memories (Goode et al., 2020), suggesting that diverse information is managed by a specific neuronal ensemble (Griffin et al., 2007; Marks et al., 2022). Thus, depending on the time when dopamine was uncaged, it could enhance specific features of the spatial memory, but how such boosting is represented at the level of neuronal ensembles remains unknown. Our experiments showed that hippocampal theta oscillations and noise levels were increased by dopamine uncaging (Fig. 5), suggesting the engagement of specific patterns of neuronal activity (Carrillo-Reid et al., 2011; Buzsaki et al., 2012). For a weak memory, the reactivation of neuronal ensembles during encoding could bias active neurons to participate in the OLM (Parsons, 2018; Guskjolen and Cembrowski, 2023), increasing their excitability and synaptic connectivity (Dragoi et al., 2003; Abraham, 2008; Mozzachiodi and Byrne, 2010; Chen et al., 2020). On the other hand, for a strong memory, longer exposure to the objects could be enough to imprint memory-related neuronal ensembles. Thus, dopamine elevation could fixate such strong OLM through increased hippocampal ensemble activation. Our results indicate that dopamine elevation enables the recurrent activation of neuronal ensembles that could have differential effects depending on memory strength.

### Dopaminergic modulation of attractor states underlying the fixation of spatial memories

The representation of the environment in the activity of hippocampal neuronal ensembles could be understood as multiple data streams that give rise to an attractor state. Thus, during the encoding stage, diverse neuronal ensembles interact generating an attractor that represents a specific environment; when an object is displaced, a remapping of the environment should take place incorporating the changes represented by new neuronal ensembles and modifying the original attractor state (Marks et al., 2022). Therefore, in the case of weak memories, the inability to recognize novel locations in our experiments could be dictated by increased variability of neuronal ensembles due to suboptimal creation of an attractor state, whereas in the case of strong memories the increased neuronal activity of a specific group of neurons successfully creates an attractor state that allows the recognition of changes in the environment. We propose that for weak memories, dopamine elevation during the encoding stage allows the formation of an attractor state by the enhancement of hippocampal neuronal ensembles related to the environment, whereas for strong memories dopamine elevation during the encoding state disables the remapping by the fixation of the original attractor state. On the other hand, when dopamine elevation occurred during the consolidation stage of strong OLMs, the inability to recognize the novel location could be explained by the creation of a different attractor state by the activation of neurons that were not related to the original environment (Colgin et al., 2008). Such mechanism differs from the nonexistent creation of an attractor state observed in the case of weak memories. It has been shown that neuronal ensemble population activity can switch between attractor states representing different spatial maps (Monasson and Rosay, 2013, 2014), suggesting that dopaminergic modulation could enable the transition between phase state regions due to changes in synaptic strength and neuronal activity.

It has been proposed that the hippocampus is optimally designed to detect events that differ from previous experiences. Thus, the hippocampus could use dopamine to modulate the relevance between internal states and external signals (Otmakhova and Lisman, 1998, 1999). Accordingly, we observed that the creation of an attractor state from weak OLMs was facilitated by dopaminergic modulation. On the other hand, our experiments showed that hippocampal dopamine elevation during the formation of strong memories fixates familiar locations, suggesting the creation of a strong attractor state that does not allow modifications. Such effect could be explained by the delayed extinction of memories (Guskjolen and Cembrowski, 2023), based on the observation that dopamine agonists reduce the depotentiation of LTP, modifying the rules of synaptic plasticity (Otmakhova and Lisman, 1998). Thus, strong memories formed in the presence of high levels of dopamine could create attractor states locked to modifications.

Therefore, we propose that dopaminergic modulation enables the transition of attractor states represented by internal activity patterns as a function of memory strength. In other words, for weak memories dopamine elevation allows the storage of attractor states, whereas for strong memories dopamine elevation potentiate existent attractor states impairing their modification by environmental changes.

### Hippocampal dopamine elevation as a memory booster

It has been proposed that the relationship between the hippocampus and the dopaminergic system could provide methods to enhance memory (O'Carroll et al., 2006; Rossato et al., 2009; Bethus et al., 2010; Papenberg et al., 2013; Urban and Gao, 2014). This is supported by the observation that increased dopamine levels transform no-learners into learners (Kempadoo et al., 2016) and facilitate LTP induction (Li et al., 2003) and that dopamine elevation could rescue memories in animal models of Alzheimer's disease (Moreno-Castilla et al., 2016). On the other hand, hyperdopaminergic levels have been related to maladaptive memories, such as the nondesired preservation of aversive memories and phobias (Osorio-Gómez et al., 2023) caused by abnormal dopamine effects on neuronal plasticity (Schultz, 2016). Accordingly, our results suggest that clinical protocols based on dopamine increase should consider the strength of memories to avoid cognitive impairments (Grace, 1991; Cools and D’Esposito, 2011). Further experiments could help to elucidate the role of dopamine in hippocampal-related pathological conditions.

## Synthesis

Reviewing Editor: Michaël Zugaro, CNRS, Collège de France, Inserm Decisions are customarily a result of the Reviewing Editor and the peer reviewers coming together and discussing their recommendations until a consensus is reached. When revisions are invited, a fact-based synthesis statement explaining their decision and outlining what is needed to prepare a revision will be listed below. The following reviewer(s) agreed to reveal their identity: Lisa Genzel, Tomonori Takeuchi.

The authors provide an interesting replication of the memory boosting effect of dopamine using a novel method (light uncaging) in mice involved in an object location task. The main findings are:

1. DA uncaging 15 min before encoding: enhanced exploration of the displaced object for weak memory encoding, and exploration of the non-displaced object for strong memory encoding.

2. DA uncaging 3 h after encoding: no impact on the 24-h test for weak memory encoding, and no evidence for preferential exploration of the displaced object for strong memory encoding.

3. DA uncaging 15 min before retrieval: no impact on the 24-h test.

4. Effects on hippocampal local field potentials: significant increases of theta, beta, and gamma bands.

The results are interesting, but several concerns arise:

Major Concerns

- Advantage of DA uncaging: What advantage does DA uncaging offer compared to micro-injection of DA receptor agonists into the dorsal hippocampus in this study?

- Physiological relevance: Considering all findings used DA uncaging, are these phenomena physiologically relevant?

- Statistics: The authors compared familiar vs novel locations, but this is inappropriate since these indices are interdependent. Instead, they should test the index for novel location against chance (vs 50% recognition index). This is likely to have a similar outcome but will be more statistically correct.

- Fig. 1C: It is unclear whether weak memory encoding actually creates a memory trace. Assessing short-term memory (e.g., 1 hr) should be considered.

- Fig. 2C: The absence of control experiments, such as bilateral irradiation without RuBi-Dopa, is concerning.

- Fig. 2F: The authors suggest that dopamine elevation during encoding impairs novelty detection. However, this result could be open to various interpretations. The authors should provide a more detailed elaboration on their interpretation to strengthen their argument.

- Line 286: The authors observed a strong preference for the displaced object in a 5-day retrieval test after strong encoding with DA uncaging. This seems to conflict with the preference for the non-displaced object in the 1-day retrieval test.

- Fig. 3C: DA uncaging 3 h after weak encoding might be outside a "grace period" for behavioral tagging (Ballarini et al., PNAS, 2009; DOI: 10.1073/pnas.0907078106), making it difficult to conclude that DA elevation during consolidation does not enhance weak memory retention.

- Fig. 3F and Line 316: The authors demonstrated that DA uncaging 3 h after strong encoding did not result in a preference for the non-displaced object, which contrasts with the findings in Fig. 2F. While the authors argue that DA elevation 3 h post-encoding impairs novelty detection, this conclusion could be influenced by other factors that have not been considered. It would be beneficial for the authors to explore and discuss alternative explanations for these results.

- Line 357: The data indicating the boosting of strong OLMs is unclear.

Minor Concerns

- Lines 55-56: The concept of "selective attention" does not fully explain the phenomenon of weak encoding followed by a novel event, nor why in Takeuchi et al (2016) memory was strengthened with DA release AFTER training (not DURING, thus not explainable by attention on task).

- Line 61: The description of optogenetic experiments might not accurately represent most studies, as they often combine pharmacological experiments to identify responsible receptors.

- Line 73: The authors' description is unclear. Although it is acknowledged that both VTA and LC project to multiple brain areas, it should be noted that some optogenetic studies have specifically targeted only the hippocampal axons originating from the VTA or LC. The authors are encouraged to clarify this aspect in the discussion.

- Line 87: Evidence for specific DA uncaging in the CA1 region is not presented.

- Methods: Why was the animal head fixed during DA uncaging? This seems like a large stressor.

- Lines 365-366, 380-382: The interpretation of the results regarding strong encoding with DA uncaging requires further elaboration.

- Lines 386-390: The authors discuss the synaptic tagging and capture theory. However, this theory suggests that dopaminergic activation enhances weak memory encoding within a 1-2 h time window, known as the 'grace period.' Therefore, this framework may not adequately explain the results presented in Fig. 3F.

- Lines 397-398: Duszkiewicz et al. (2019) proposed that novel events facilitate memory consolidation rather than promote memory formation.

- Lines 402-405: The conclusion regarding novelty detection and the fixation on familiar information needs more precise evidence and explanation.

- Fig. 5: Show whole power spectrum since effect seems unspecific of bands but instead a wide-band effect.

## Author Response

We thank the Reviewers and Editor for their helpful comments and suggestions that we believed have strengthened the impact of our study. We addressed all the recommendations and change the text accordingly including additional experiments, figure panels, statistical analyses, and an elaborated discussion. We also added some references to support our conclusions in the updated version of the manuscript. All the changes to the manuscript are highlighted in red in the Track version.

For clarity, our responses to reviewers are highlighted in bold style.

Comments to the manuscript and responses:

Major Concerns - Advantage of DA uncaging: What advantage does DA uncaging offer compared to micro-injection of DA receptor agonists into the dorsal hippocampus in this study? Response: We added new lines in the introduction describing the advantages of caged compounds compared to microinjection of DA receptor agonists (lines 84-94). Briefly, the main advantage of caged DA is that its release can be controlled temporarily and spatially. The release of dopamine occurs in the volume where the light is incident and the uncaging is dependent on the characteristics of the light source that can be precisely modeled (Stujenske et al., 2015). On the contrary, the microinjection of DA receptor agonists is subject to diffusional delays that also involve a concentration gradient that varies from the site of the injection throughout the diffused volume. On the other hand, it has been shown from Parkinson's Disease studies that direct dopamine injection represents an issue due to rapid oxidation of DA (Horne et al., 1989). Moreover, it is also known that DA receptor agonists could cause non-desired side effects such as hallucinations or compulsive behaviors (Blandini and Armentero, 2014; Talati et al., 2009; Voon et al., 2011) that could affect the memory task used in our experiments.

- Physiological relevance: Considering all findings used DA uncaging, are these phenomena physiologically relevant? Response: In the present study we demonstrated the proof of principle that DA uncaging could be used to study memory processes. We consider that our results are physiologically relevant in the context of drugs that alter DA levels, given the fact that FDA approved dopaminergic agonists are routinary used clinically to treat movement related disorders such as Parkinson's disease. On the other hand, it is known that DA replacement therapies increase DA levels in all the brain, and that the use of DA therapy has been suggested for Alzheimer's Disease. Moreover, it has been shown that DA elevation could contribute to recurrent behaviors characterized by fixated memories that are observed in substance-dependent users. However, how dopamine elevation in the hippocampus could differentially affect weak or strong memories at different learning states has not been studied. Thus, understanding how hippocampal dopamine elevation could be related to the enhancement or fixation of memories is physiologically relevant. We consider that our findings are a first step to understand hippocampal memory processes that are DA dependent in normal and pathological conditions. We added new lines in the introduction emphasizing the physiological relevance of our results (lines 77-83).

- Statistics: The authors compared familiar vs novel locations, but this is inappropriate since these indices are interdependent. Instead, they should test the index for novel location against chance (vs 50% recognition index). This is likely to have a similar outcome but will be more statistically correct.

Response: We appreciate the Reviewer comment, in the updated version of the manuscript we used the suggested statistical analysis to test the recognition index for the novel location against 50% chance levels and modified accordingly the figures. As the Reviewer mentioned the outcome of the new statistics is similar to the reported in the previous version of the manuscript.

- Fig. 1C: It is unclear whether weak memory encoding actually creates a memory trace. Assessing short-term memory (e.g., 1 hr) should be considered.

Response: It has been previously shown in mice that a weak OLM protocol actually creates a memory trace that can be recovered after 1hr as the reviewer suggests (Murai et al., 2007). However, to corroborate such results and directly address the Reviewer concern we included data from a new group of mice (Figs 1A, 1B &1C) showing that 5 minutes exposure to two identical objects (weak OLM) created a memory trace that allow mice to recognize the novel location of the object after 1 hr, confirming that the weak memory OLM protocol creates a short-term memory trace.

- Fig. 2C: The absence of control experiments, such as bilateral irradiation without RuBi-Dopa, is concerning.

Response: We appreciate this Reviewer's observation. We included in the updated version of the manuscript data from a new group of mice showing that bilateral blue light irradiation has no effect on OLMs (lines 308-313).

- Fig. 2F: The authors suggest that dopamine elevation during encoding impairs novelty detection. However, this result could be open to various interpretations. The authors should provide a more detailed elaboration on their interpretation to strengthen their argument.

Response: We agree with the Reviewer about that our results could be open to various interpretations and that they not necessarily demonstrate the impairment of novelty detection. We modified such claim throughout the manuscript to state that in our experiments, dopamine elevation impairs the increased exploration of the object in the novel location. Besides, we also modified the discussion to further explain the rationale of the paradoxical effect of DA between weak and strong memories.

- Line 286: The authors observed a strong preference for the displaced object in a 5-day retrieval test after strong encoding with DA uncaging. This seems to conflict with the preference for the non-displaced object in the 1-day retrieval test.

Response: We appreciate this comment that challenged us to explain better our rationale for this apparent conflict in our results. To clarify this issue, we added a new paragraph in the discussion of the updated version of the manuscript (Dopaminergic modulation of attractor states underlying the fixation of spatial memories. Lines 480-529).

- Fig. 3C: DA uncaging 3 h after weak encoding might be outside a "grace period" for behavioral tagging (Ballarini et al., PNAS, 2009; DOI: 10.1073/pnas.0907078106), making it difficult to conclude that DA elevation during consolidation does not enhance weak memory retention.

Response: It is worth to notice that in the reference mentioned by the reviewer (Ballarini et al., 2009) rats were exposed to novel environments evoking the increase of activity in LC whose projections are not exclusive to the dorsal hippocampus which differs from our experiments. However, to address the reviewer concern we included additional data from another experimental group where we uncaged DA 1 hr (Figures 3A, 3B &3C) after the encoding of a weak OLM protocol. We found that even at such time window DA elevation was not able to enhance weak OLMs. We added a new paragraph in the discussion suggesting that the weak OLM protocol in mice could not be enough to engage plasticity related proteins that could be modulated by dopamine, explaining the lack of effect of DA elevation observed after the encoding of a weak OLM in our experiments. It is worth to notice that spatial memory mechanisms could differ between mice and rats as has been highlighted by Kentros et al., 2004. Our new set of experiments demonstrates that DA elevation after the encoding of a weak OLM does not enhance weak memories.

- Fig. 3F and Line 316: The authors demonstrated that DA uncaging 3 h after strong encoding did not result in a preference for the non-displaced object, which contrasts with the findings in Fig. 2F. While the authors argue that DA elevation 3 h post-encoding impairs novelty detection, this conclusion could be influenced by other factors that have not been considered. It would be beneficial for the authors to explore and discuss alternative explanations for these results.

Response: We appreciate this suggestion. We agree with the Reviewer that our data not necessarily demonstrates impairment of novelty detection. It is worth to notice that DA elevation during the consolidation of a strong OLM affects spatial memories in the absence of contextual information, supporting the observation of differential effects between DA uncaging during encoding compared to DA uncaging during consolidation of long-term memories. We added a new paragraph in the discussion elaborating our alternative explanations for these results in terms of attractor dynamics.

- Line 357: The data indicating the boosting of strong OLMs is unclear.

Response: We modified the results section and Figure 5 to include further analyses to clarify our results and interpretation of the data.

Minor Concerns - Lines 55-56: The concept of "selective attention" does not fully explain the phenomenon of weak encoding followed by a novel event, nor why in Takeuchi et al (2016) memory was strengthened with DA release AFTER training (not DURING, thus not explainable by attention on task).

Response: We rephrased the paragraph to clarify our statement (lines 51-55). It is worth to notice, that the spatial learning task in Takeuchi et al., 2016 mentioned by the Reviewer, implies a spatial memory that is dependent on a rewarded location in a familiar arena. Thus, the reward obtained by mice could have evoked increased DA levels in the hippocampus during encoding. Accordingly, Takeuchi et al., 2016 showed in Extended Data Figure 1d that increased reward during encoding even in the absence of optogenetic stimuli resulted in a persistent memory 24 hours later.

- Line 61: The description of optogenetic experiments might not accurately represent most studies, as they often combine pharmacological experiments to identify responsible receptors.

Response: We modified the text to clarify our statement (lines 60-65).

- Line 73: The authors' description is unclear. Although it is acknowledged that both VTA and LC project to multiple brain areas, it should be noted that some optogenetic studies have specifically targeted only the hippocampal axons originating from the VTA or LC. The authors are encouraged to clarify this aspect in the discussion.

Response: We modified the introduction to clarify our statement.

- Line 87: Evidence for specific DA uncaging in the CA1 region is not presented.

Response: For the light stimulation parameters used in our experiments (fiber optic of 400 µm diameter, NA 0.39 , 4mW, 20% duty cycle, 20 Hz, 475nm) the light propagation englobes a depth distance <300 µm and a radial distance <200 µm at the tip of the optical fiber (Stujenske et al., 2015), beyond such propagation distances light intensity drops to less than 1%, and it has been shown that low light intensity power is not able to uncage RuBi-Dopa in vivo (Zamora-Ursulo et al., 2023).

- Methods: Why was the animal head fixed during DA uncaging? This seems like a large stressor.

Response: We observed that an optical fiber attached to mice reduced their mobility. We performed preliminary experiments (not shown) with the fiber optic attached to the skull, and we observed that the total exploration time of the objects was reduced compared to mice without an optical fiber attached to the skull. Under such conditions mice explored similar to chance levels the familiar location and the novel location in the retrieval stage for the strong OLM protocol. It is worth to notice that head-fixed mice can adapt to the head fixation and learn to perform tasks even with the head attached to a recording setup. In the methods section we added a new paragraph to clarify that mice were handed and exposed to head-fixation conditions for several days before the task protocol dismissing possible stressor issues.

- Lines 365-366, 380-382: The interpretation of the results regarding strong encoding with DA uncaging requires further elaboration.

Response: We further elaborate the rationale regarding the results of DA uncaging during the encoding of a strong OLM (lines 480-529).

- Lines 386-390: The authors discuss the synaptic tagging and capture theory. However, this theory suggests that dopaminergic activation enhances weak memory encoding within a 1-2 h time window, known as the 'grace period.' Therefore, this framework may not adequately explain the results presented in Fig. 3F.

Response: It is worth to notice that lines 386-390 in the previous version of the manuscript referred to the strong OLM protocol. We now included more data showing that for the weak OLM protocol, DA uncaging was not able to enhance weak memory encoding within a 1-2h time window. We also added additional experiments where we used a D1-like receptor antagonist during dopamine uncaging in the encoding stage of a weak OLM, showing that if the retrieval stage is performed after 1 hr, mice explored more the displaced object demonstrating that the short-term memory induced by the weak OLM protocol doesn't engage plasticity related proteins and therefore cannot be subjected to DA actions.

- Lines 397-398: Duszkiewicz et al. (2019) proposed that novel events facilitate memory consolidation rather than promote memory formation.

Response: We rephrased our statement (lines 448-450) to highlight that dopamine receptor activation engages plasticity related proteins that could transform transient LTP to persistent LTP as proposed by Duszkiewicz et al. 2019.

- Lines 402-405: The conclusion regarding novelty detection and the fixation on familiar information needs more precise evidence and explanation.

Response: We appreciate this comment. Throughout the text we have modified our statements about the role of DA uncaging regarding novelty detection, stating that in our experiments DA uncaging impairs the OLM task. We agree that the effect regarding novelty detection requires different experiments that are beyond the goal of the current manuscript. On the other hand, we also added a new paragraph in the discussion explaining the possible neuronal ensemble mechanism underlying strong memory fixation using the framework of attractor states.

- Fig. 5: Show whole power spectrum since effect seems unspecific of bands but instead a wide-band effect.

Response: We appreciate this suggestion. In the updated version of the manuscript, we performed different analyses on the data and modified the Figure 5 including the power spectrum as requested. We observed that as suggested by the reviewer the increase in gamma and beta bands was caused by a wide-band effect. However, after the subtraction of the aperiodic component (Donoghue et al., 2020) we still observed a significant increase in the theta band evoked by dopamine elevation. In the updated version of the manuscript, we modified the text accordingly to describe the new analyses (lines 394-424). Importantly, such results further confirm our original interpretation of the data suggesting that DA uncaging could promote the synchronization of neuronal ensembles in CA1.
